# A 1–7 year retrospective follow-up on consecutively placed 7-mm-long dental implants with an electrowetted surface

**DOI:** 10.1186/s40729-018-0136-4

**Published:** 2018-08-23

**Authors:** Paul S. Rosen, Herman Sahlin, Rudolf Seemann, Ari S. Rosen

**Affiliations:** 1 Clinical Professor of Periodontics, Baltimore College of Dental Surgery, University of Maryland Dental School, Baltimore, MD USA; 2 Private Practice limited to Periodontics and Dental Implants, 907 Floral Vale Boulevard, Yardley, PA 19067 USA; 3 Neoss Ltd, Gothenburg, Sweden; 4 University Clinic of Craniofacial, Maxillofacial and Oral Surgery, Vienna, Austria; 5 University of Delaware Newark, Delaware, USA

**Keywords:** Short implants, Cumulative survival rate, Implant stability, Electrowetted surface

## Abstract

**Background:**

This retrospective consecutive case series study was performed to determinate the survival rate and implant stability of short (7 mm length) dental implants with an electrowetted hydrophilic surface that were in function from 1 to 7 years.

**Methods:**

A retrospective chart review identified and evaluated 86 consecutively placed 7-mm-long dental implants (ProActive, Neoss Ltd., Harrogate, England) in 75 patients. Analysis was performed for implant survival as well as implant stability, as measured by insertion torque (IT) and resonance frequency analysis (RFA).

**Results:**

Clinical follow-ups were performed from 1.0 to 7.0 years after implant placement (mean 4.0 ± 2.1 years). Two implants failed prior to loading resulting in a 5-year cumulative survival rate (CSR) of 97.7%. An additional late failure occurred at 60 months post-loading for a 7-year CSR of 94.8%. Mean insertion torque was 30.1 ± 7.4 Ncm and mean RFA at insertion was 73.6 ± 8.1 ISQ. Follow-up RFA measurements suggested that the achieved primary stability was maintained throughout the healing phase.

**Conclusion:**

The present study demonstrates that treatment with short implants can be a predictable treatment option with high survival rate in sites with limited available bone.

## Background

In the past decades, the osseointegration rate of dental implants has dramatically increased, particularly in sites of softer dental bone, which may be attributed to the introduction of moderately roughened surfaces [1, 2]. Moreover, because of this increase in success, clinicians have attempted to push the envelope and place implants into sites that may provide a greater challenge as they wish to meet their patients’ oral health needs. Short dental implants have been explored in various indications such as close proximity to the inferior alveolar nerve or at an area where there is a highly pneumatized sinus floor to potentially reduce the time associated with the surgery and the prolonged healing period attendant with complex grafting/surgical procedures that would be needed for placing longer dental implants. Moreover, these grafting procedures may introduce certain post-operative morbidities not seen with standard dental implant placements. Three randomized controlled studies comparing short to longer dental implants that were placed into augmented sites found no significant differences in implant survival between the groups [3–5], suggesting comparable clinical outcomes for short and longer implants up to 3 years after implant surgery. Several systematic reviews/meta-analyses on the use of short implants have also demonstrated similar results [2, 6–8]. Esposito et al. performed a meta-analysis that demonstrated a non-significant trend of more implant failures (OR = 5.74) and more complications (OR = 4.97) in the group of implants placed into vertically augmented sites versus the short dental implant group [6]. Pommer et al. performed a systematic review and found that short implants with a machined surface had a higher early failure rate than longer implants with a machined surface (OR = 2.2) [2]. However, with rougher surfaces, no difference was seen (OR = 1.1). Another systematic review and meta-analysis comparing short and long implants found that short implants are as predictable as long implants (CSR 88.1 vs. 86.7%), but the failures tend to occur at an earlier time point [8]. A recent systematic review by Lemos et al. found that short implants (8 mm and shorter) were as predictable as standard implants (> 8 mm). However, implants shorter than 8 mm showed lower survival rates than standard implants (RR = 2.05) [7].

The aim of this retrospective consecutive case series study was to investigate implant survival rate and analyze possible factors affecting the survival of short implants placed in one surgical practice focused on implantology and periodontology in a temporal cohort.

## Methods

A retrospective study on short 7 mm hydrophilic implants from a single center was conducted in a private practice limited to periodontics and surgical dental implant placements from one of the authors, PSR (Yardley, Pennsylvania, USA). An exhaustive chart review identified 75 patients for analysis that were treated with 86 short (7 mm) implants during a 5-year period (September 1, 2009, to November 1, 2014). The study was performed in compliance with the Declaration of Helsinki. Data collection was performed in such a manner that subjects could not be identified, and therefore, it was exempt from IRB review according to Federal Regulation 45 CFR 46.101(b).

**Fig. 1:**
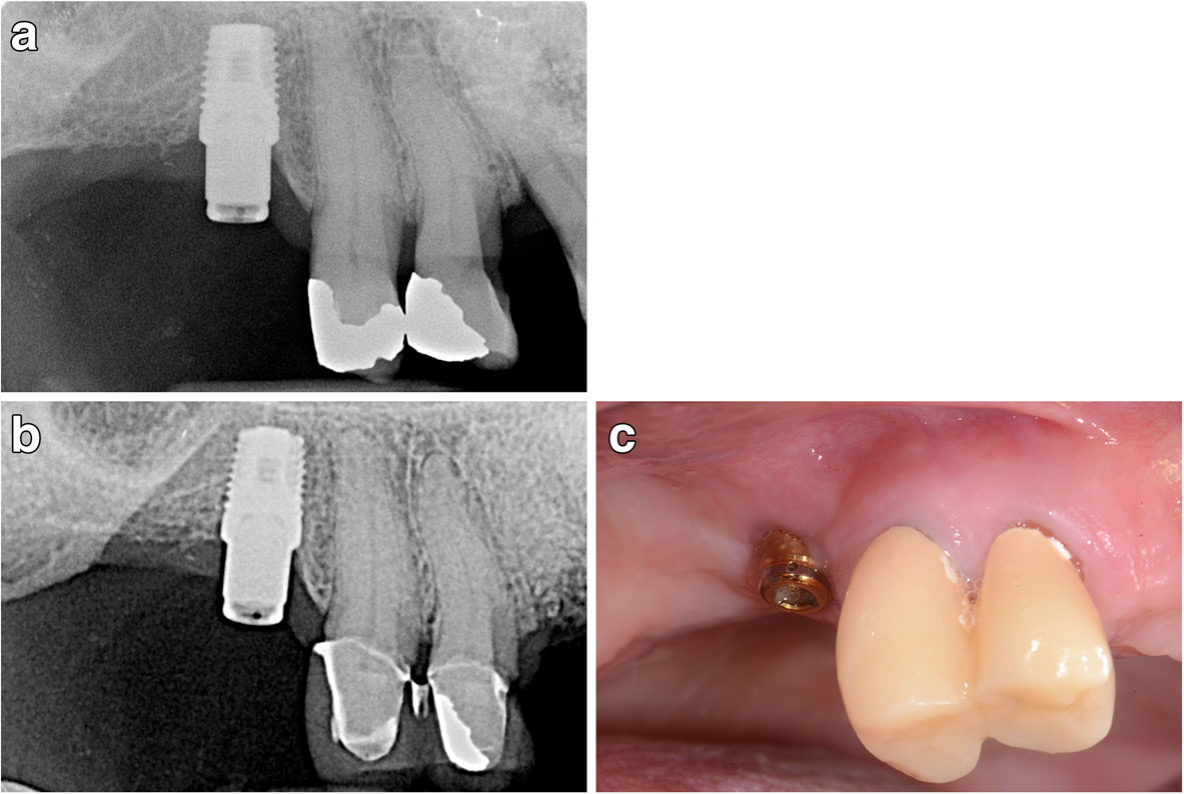
**a** Initial radiograph exposed at abutment installation on a 5-mm-wide by 7-mm-length dental implant used to help support a removable partial denture for a 71-year-old Caucasian male. **b** Radiograph of the area taken at 82 months demonstrates good bone stability. **c** Clinical image of the area 82 months later. Soft tissue remains healthy. The two teeth anterior are in the process of receiving a two-unit fixed splint

**Fig. 2:**
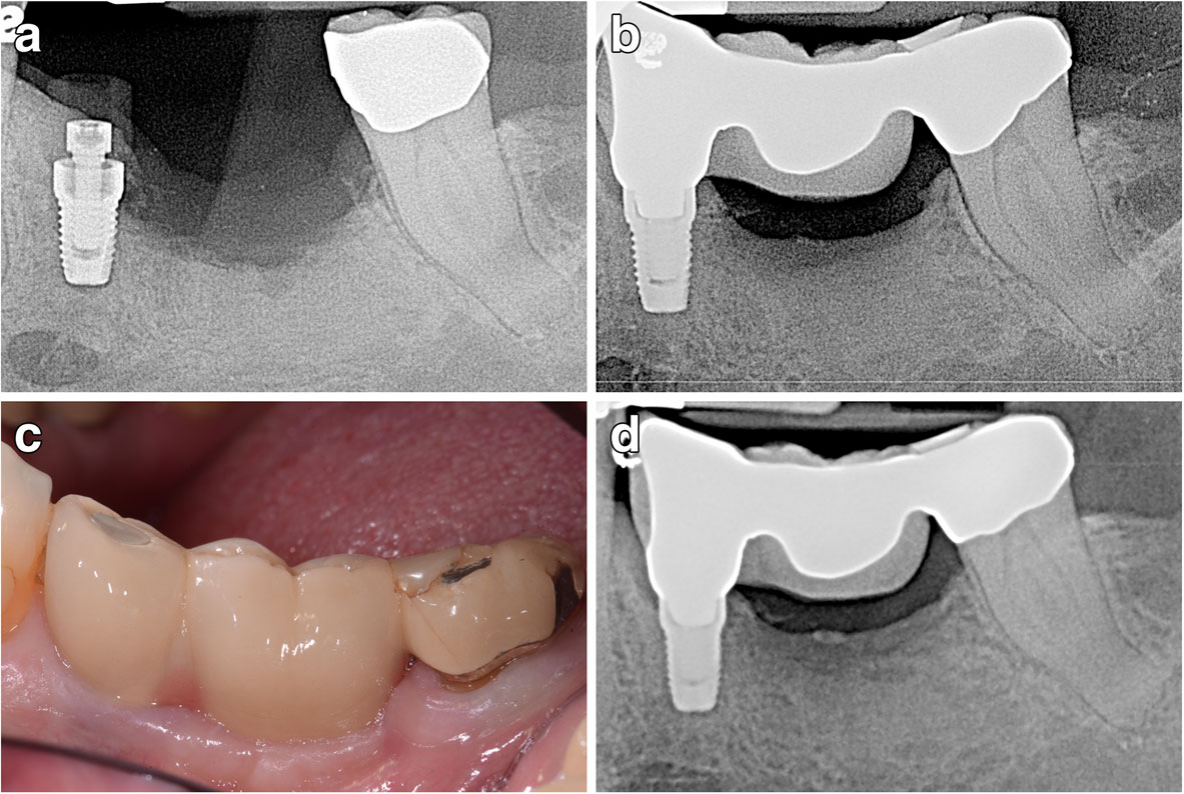
**a** Radiographic image of a 3.5-mm-wide by 7-mm-length dental implant at the time of its placement at the mandibular left second premolar in a 63-year-old Caucasian female. **b** Radiographic image of a three-unit fixed partial denture upon its initial placement. The dental implant is the anterior abutment with the prosthesis screw retained to it. The distal abutment is the mandibular left second molar. A coping has been placed in the event of intrusion. **c** Clinical image of the three-unit fixed partial denture at 58 months post-loading. Soft tissue health has been maintained around both the tooth and the dental implant. **d** Radiograph exposed at 58 months demonstrating the steady state of bone on both the dental implant and the natural tooth abutments. There is no suggestion of any intrusion occurring with the mandibular left second molar

All implants had a hydrophilic electrowetted surface (ProActive™, Neoss Ltd., Harrogate, UK) and were placed according to the manufacturer’s instructions using either a one-stage protocol with a transgingival healing abutment or a two-stage submerged healing for 5–8 weeks. Implants were loaded after 2 to 6 months (Figs. 1 and 2).

Postoperative management included the use of amoxicillin 875 mg twice daily for 7 days along with the use of 0.12% chlorhexidine gluconate mouthrinse topically applied twice daily for at least the first 28 days. If the patient was allergic to amoxicillin, then either clindamycin 150 mg taken four times daily for 7 days or azithromycin 500 mg taken on the first day followed by 250 mg per day for the next 4 days was substituted. For pain management, patients used ibuprofen 600–800 mg up to four times per day or acetaminophen with codeine # 3 taken every 4–6 hours if non-steroidal anti-inflammatory agents could not be taken.

Suture removal took place at 14 ± 3 days post-implant insertion. RFA was repeated at varying time points depending upon the data recorded at initial placement. Due to the study design (retrospective analysis of patient records compiled in a private practice setting), standardized annual radiographs were not taken. Timing and frequency of radiographic examination varied depending upon when the patients returned back to the surgeon’s clinic and thus did not allow for a systematic analysis as part of the study.

Baseline parameters, both patient- and implant-related, as well as follow-up parameters (implant survival, follow-up time, and resonance frequency analysis) were collected from a review of the patient records.

### Statistics

The main study parameters (principal outcome parameters) were defined to be implant loss and follow-up time. The cumulative survival rate was estimated. The influence of several factors on survival was tested in Cox proportional hazard models: age, sex, smoking, diabetes, osteoporosis, jaw, implant position, bone type, implant diameter, insertion torque, RFA at insertion, healing protocol, and prosthetic restoration. In case of continuous variables (age, torque, and RFA), an optimal cutoff value for implant failure prediction was computed based on the Youden’s index [9]. Finally, all factors were added to a multivariate Cox proportional hazard model to correct for multiple testing.

## Results

The chart review identified 86 placed implants in 75 patients. Table 1 summarizes the patient demographics of the 75 patients. Patients ranged in age from 29 to 88 years with a mean of 61.0 ± 12.5 years. Twenty-seven of the patients were males and 48 were females. Table 2 summarizes the implant and site-related information of the 86 placed implants. Mean insertion torque was 30.1 ± 7.4 Ncm (range 10–50), and mean RFA at the time of implant placements was 73.6 ± 8.1 ISQ (range 35–87).

**Table 1:** Patient demographics

		Number	Percent
Age (years)	20–29	1	1.3
	30–39	4	5.3
	40–49	6	8.0
	50–59	19	25.3
	60–69	27	36.0
	70–79	12	16.0
	80–89	6	8.0
Sex	Female	48	64.0
	Male	27	26.0
Smoker	No	69	92.0
	Yes	6	8.0
Diabetes	No	71	94.7
	Yes	4	5.3
Osteoporosis	No	66	88.0
	Osteopenia	5	6.7
	Osteoporosis	4	5.3
Penicillin-VK allergy	No	65	86.7
	Yes	10	13.3

**Table 2:** Implant and site-related specifications

		Number	Percent
Jaw	Maxilla	60	69.8
	Mandible	26	30.2
Position	Anterior	2	2.3
	Posterior	84	87.7
Implant diameter	3.5 mm	4	4.7
	4.0 mm	14	16.3
	4.5 mm	13	15.1
	5.0 mm	21	24.4
	5.5 mm	32	37.2
	6.0 mm	2	2.3
Site type	Healed site	48	55.8
	Immediate extraction	31	36.0
	Implant replacement	1	1.2
	Sinus	6	7.0
Bone quality (Lekholm/Zarb)	Type 1	4	4.7
	Type 2	54	62.8
	Type 3	26	30.2
	Type 4	2	2.3
Insertion torque (Ncm)	10–19	6	7.0
	20–29	14	16.3
	30–39	59	68.6
	40–49	4	4.7
	50	3	3.5
Implant stability at placement (ISQ)	30–39	1	1.2
	40–49	1	1.2
	50–59	1	1.2
	60–69	17	19.8
	70–79	44	51.2
	80–89	22	25.6
Surgery	One-stage	44	51.2
	Two-stage	42	48.8
Type of restoration	Crown	48	55.8
	Bridge	35	40.7
	Locator	3	3.5

Of the 86 implants, 84 (97.7%) successfully osseointegrated and were restored. Clinical follow-ups were recorded up to 7.0 years after implant placement with a mean follow-up time of 4.0 ± 2.1 years. There were three (3.5%) failed implants recorded in three (4.0%) different patients. Specifications on the failures are given in Table 3. The implant-based cumulative survival rate was 97.7% after 1 year, 97.7% after 5 years, and 94.8% after 7 years (Table 4).

**Table 3:** Specification of failed implants (three implants in three patients)

Sex	Age	Smoker	Risk factors	Position	Implant diameter	Insert. torque	ISQ at insert.	Time of failure
Male	75	No	Diabetes	Maxillary premolar	5.5 mm	20	75	0 months
Male	73	No	No	Maxillary premolar	4.5 mm	32	80	60 months
Female	77	No	Osteoporosis	Mandibular molar	5.0 mm	32	70	0 months

**Table 4:** Implant survival, life table analysis

Interval	Implants	Failed	Not followed	CSR (%)
Insertion to 1 year	86	2	3	97.7
1 to 2 years	81	0	19	97.7
2 to 3 years	62	0	6	97.7
3 to 4 years	56	0	6	97.7
4 to 5 years	50	0	16	97.7
5 to 6 years	34	1	16	94.8
6 to 7 years	17	0	15	94.8
7 years	2	–	–	–

Subgroup analysis was performed to identify if any factors had an effect on implant survival, i.e., if they were risk factors. The analysis failed to reveal any significant differences between subgroups, i.e., *p* > 0.05 for all tested parameters. Hence, none of the factors were identified as a risk factor. The mean insertion torque was 30.1 ± 7.5 Ncm in successful implants and 28.0 ± 6.9 Ncm in the three that failed. The mean ISQ value was 73.6 ± 8.2 in the successful implant group and 75.0 ± 5.0 in the failed implants group.

RFA measurements during the healing phase, i.e., up to 16 weeks, are presented in Table 5. The high mean RFA value at insertion (73.6 ± 8.1 ISQ) was well maintained up until 16 weeks, indicating maintained implant stability at a high level throughout the healing period.

**Table 5:** Resonance frequency analysis

Time	ISQ	*n*
Implant insertion	73.6 ± 8.1	86
1–4 weeks	79.4 ± 4.1	28
5–8 weeks	77.3 ± 5.0	34
9–12 weeks	74.9 ± 5.6	20
13–16 weeks	73.3 ± 4.4	11

## Discussion

This retrospective study is the first to look at short dental implants with a hydrophilic electrowetted surface. The survival data suggest that this treatment is a viable option to care. In a systematic review that identified 13 studies on implants shorter than 10 mm, the CSR from the individual studies ranged from 80 to 100% with a combined CSR of 98.3% after 5 years, 94.8% after 6 years, and 88.1% after 14 years [8]. It should be noted that the CSR of the current study is comparable to the combined CSR for the identified 13 studies although the majority (56%) of the reported short implants in the reviews were 8–9 mm, i.e., longer than the implants followed in the current study. Another systematic review that looked only at comparative studies between short and long implants found a combined failure rate of 4.6% (32 of 700) for implants shorter than 8 mm after an average of 1.8 years (range 0.25–5 years) [7]. The present study demonstrated a slightly lower failure rate of 3.5% (3 of 86) after an average of 4.0 years. The cumulative survival rate for the current study (97.7% after 5 years, 94.8% after 7 years) was in the upper end of the range reported in these other studies on short implants, indicating treatment outcomes well in line with or even better than the majority of short implant studies reported in the literature. In current scientific literature, a 13.4-year CSR of long implants was reported to be 94.6% [10]. This CSR for standard dental implants is at the same level as the 7-year survival rate for the 7-mm implants in the current study.

Since the treatment protocol is kept simple, it might be preferred by both patients and clinicians to treatments that involve more invasive techniques such as nerve transposition, guided bone regeneration, and sinus elevation procedures. The success of the reported treatment approach might in part be due to the use of implants with a diameter wider than 4 mm (68 of 86 implants). The wider implants were placed into sites with significant lateral bony dimensions to allow for their placement without over-preparing the sites and compromising residual bone width. This helped to maximize the implant surface and hence the implant-bone interface needed for successful osseointegration.

A wide distribution in implant insertion torque (10–50 Ncm) was seen in the study. This reflects the variety of clinical situations in which the implants were placed. Assessments were made by using both the RFA value and the insertion torque as to first whether an implant should have been left to heal in the first place and if so, how this would be best accomplished, i.e., through its submergence with a cover screw or by transgingival exposure with a healing abutment.

The study aimed at identifying factors affecting survival of 7-mm electrowetted-surfaced implants. In the temporal cohort of this study, none of the tested parameters showed any significance. This means that none of the factors were identified as risk factors for short implants. However, in a study cohort with only three implant failures in total, the possibility of missing a true risk factor is high for pure mathematical reasons. Therefore, larger patient populations are needed to properly identify the risk factors.

In the current study, two early losses occurred within 1 year. This is in line with a systematic review on 690 short (6 mm) implants, which reported that 76% of all losses were early [11]. Two of the three patients that lost implants had comorbidities such as diabetes (type 2) and osteoporosis. Diabetes, although known as a risk factor for periodontitis, has not been confirmed to affect implant survival [12]. Osteoporosis is also not confirmed as a risk factor, but at least has demonstrated a trend [13]. Diabetes and osteoporosis were not significant factors in this study. This may well be related to the small number of patients who had these systemic diseases thus providing a small overall effect.

The maxilla is commonly seen as having less favorable bone quality than the mandible [14]. Therefore, one concern would be that 7-mm-long implants placed in the maxillary arch might be at higher risk for failure than those placed in the mandible. In the current retrospective, the survival rate was similar between the two arches. Hence, the notion that one arch versus the other would be at greater risk with this particular short dental implant appears to hold no validity. A larger prospective study would need to be undertaken with more sites/clinicians placing the implants to determine if this finding is generalizable.

Thirty-six percent of the implants in the study were placed in extraction sockets. Provided that sufficient initial implant stability is achieved, there should be no additional risk factors compared to implants in healed sites. Studies have shown no difference in marginal bone remodeling between immediately placed and delayed implants [15].

The biggest limitation of the current study is its very nature as retrospective single-center study with a limited sample size. The included patients received 7-mm-long implants. There is no control group with longer dental implants of the same surface to allow for direct comparisons to be made.

## Conclusion

The current retrospective consecutive case series study provides preliminary data that treatment with 7-mm-length short implants with a hydrophilic electrowetted surface is a reasonable approach in sites with limited vertical bone dimension. It adds to the body of evidence supporting short implant use for compromised sites. The success seen might be attributed to the larger implant diameters that were used to increase surface area. Further prospective studies need to be performed in larger patient population and with multiple centers to determine the generalizability of this approach.

## Abbreviations

CSR: Cumulative survival rate
ISQ: Implant stability quotient
IT: Insertion torque
OR: Odds ratio
RFA: Resonance frequency analysis
RR: Relative risk
